# Poststroke Outcomes

**DOI:** 10.1155/2014/828435

**Published:** 2014-10-14

**Authors:** Bruce Ovbiagele, Steve Kautz, Wayne Feng, DeAnna L. Adkins

**Affiliations:** ^1^Department of Neurology and Neurosurgery, College of Medicine, Medical University of South Carolina, Charleston, SC 29425, USA; ^2^Department of Health Science & Research, College of Health Professions, Medical University of South Carolina, Charleston, SC 29425, USA; ^3^Ralph H. Johnson VA Medical Center, Charleston, SC 29425, USA; ^4^Department of Neuroscience, College of Medicine, Medical University of South Carolina, Charleston, SC 29425, USA

Stroke is a leading cause of death and disability [1, 2]. While stroke mortality rates are decreasing due to improved medical treatment of the complications caused by acute stroke, the number of individuals living with the residual effects of stroke is rising [3]. Currently, over 75% of patients survive a first stroke, and, of these individuals, 25% are left with a minor disability and 40% experience moderate-to-severe disabilities [4]. Furthermore, stroke patients are at high risk for future vascular events, including recurrent stroke, putting them at a greater risk of death and further disability [5]. With growing numbers of stroke survivors, there is an urgent need to improve our understanding of the short- to long-term recovery process after stroke and to identify avenues for developing efficacious therapeutic strategies to enhance poststroke outcomes [6]. Government research funding agencies, like the National Institute of Neurological Diseases and Stroke (NINDS), and nongovernmental research funding organizations, like the American Heart Association (AHA), have recognized the need for prioritizing poststroke outcomes research by developing strategic plans to explore ways in which the brain affected by stroke or endangered by risk factors can preserve, protect, or recover function and supporting consortia of multidisciplinary investigators and facilities conducting collaborative investigation into stroke regeneration, resilience, and secondary prevention [7, 8].

This special issue was developed to shed light on the various factors affecting the central and peripheral nervous system, which influence prognoses following a stroke, as well as to portray promising new poststroke treatment modalities. In the systematic qualitative review conducted by C. Ellis and colleagues, they found evidence of a racial disparity in poststroke functional outcomes, with people of Black race, who have the highest risk for stroke incidence and mortality [9], having poor outcomes after a stroke compared to their non-Hispanic White counterparts. Among 355 ischemic stroke patients who received thrombolytic therapy, D. Nathanson et al. observed that women experienced better recovery outcomes at 3 months after stroke than men and that lower diastolic blood pressures in women may contribute to this gender difference. Findings from both studies highlight a need to properly establish the contributors to demographic disparities in stroke outcomes and implement interventions to equitably enhance favorable sequela after stroke.

M. Poorjavad et al. performed a systematic review on the use of surface electric stimulation intensity and electrode placements to treat swallowing disorders after stroke, recommending that additional research should focus on better discrimination of the underlying neurophysiologic effects of these therapeutic methods on swallowing function. In a phase I study, A. Sharma and team evaluated the feasibility and potential effects ofautologous bone marrow mononuclear-cell intrathecal transplantation in chronic stroke patients, noting that relatively younger patients, those who underwent therapy within 2 years of stroke, and those with ischemic versus hemorrhagic strokes showed better recovery. On the other hand, D. K. Rose and colleagues found that low-frequency rTMS stimulating contralesional hemisphere did not augment upper extremity motor ability in a population of individuals with chronic stroke. They suggest that the chronicity of their cohort (on average ≥5 years from stroke onset) and degree of upper motor impairment may have led to an inability of rTMS to provide robust effects.

Regaining the ability to locomote after a stroke is a complex process. In particular, several factors directly or indirectly influence an individual's capacity to walk after stroke, including ankle muscle weakness, balance issues, and other factors. Five distinct articles investigate poststroke walking from different perspectives. C. K. Balasubramanian and her group examined conceptual challenges for clinical measurement of walking adaptability and summarized the current state of clinical assessment for walking adaptability in a systematic review, calling for development of a comprehensive well-tested clinical assessment tool for measuring walking adaptability. While many people think improvement in balance should lead to better long-distance walking function after stroke, in their paper, L. N. Awad and colleagues challenge this premise, finding that improving balance may not necessarily be sufficient to improve long-distance walking function. J. W. Ramsay et al. contributed two papers to this journal issue, by carefully analyzing the muscular morphology and architecture in both dorsiflexors and plantar flexors using combined imaging modalities (MRI and ultrasound). In both papers, his team found no major differences between the affected and unaffected sides, or compared to healthy controls, concluding that ankle muscle weakness is mainly neural in origin and is possibly secondary to muscle activation failure. The same research group also has another article (led by Knarr) in which musculoskeletal simulations of individuals after stroke with subject-specific muscle force and activation data were created. This study indicated that after a stroke, subject-specific muscle force and activation data may improve the ability of musculoskeletal simulations to precisely predict muscle coordination.

Spasticity hinders stroke motor recovery and affects global outcomes [10]. This is quantitatively demonstrated in the paper by S. R. Belagaje and colleagues in which presence of spasticity was associated with a worsening of mRS by average of 0.4 at 3 months after stroke. However, the pathological mechanisms of poststroke limb spasticity are not clear. S. Pundik et al. used fMRI and behavioral assessment to examine stroke patients receiving motor learning and spasticity therapy. They found that greater baseline spasticity correlated with higher fMRI activation in the ipsilesional thalamus, and, after therapy, greater mitigation of spasticity correlated with enhanced fMRI activation in the contralesional primary motor, premotor, primary sensory, and associative sensory regions, even when controlling changes in motor function. They concluded that the contralateral motor region may play a role in spasticity and represent a novel target for treatment.

The widely used NIH stroke scale did not correlate well with validated measures of upper extremity functional impairment, and so B. Hand and colleagues recommend that its use should be restricted only to acute stroke studies and clinical settings with the objective of reporting stroke severity and not to studies of stroke recovery. By using Rasch analysis, J. S. Sabari et al. redesigned the two scales that comprise the hand function items on the motor assessment scale. The developed hand movements and hand activities items each measure a unidimensional construct and can reliably measure stroke patients with different levels of hand functional impairments.

In conclusion, several opportunities exist to expand the scientific underpinnings and therapeutic options pertaining to poststroke outcomes. Periodic journal issues wholly dedicated to covering the state of the science in this research area are crucial for identifying gaps, stimulating ideas, and planning for future evidence-based treatments. That was the objective of this particular issue. Fortunately, improving poststroke outcomes is now clearly a priority item on the agenda of policy makers at various levels of medical research funding and healthcare delivery. We sincerely hope that these ongoing endeavors will lead to major breakthroughs in stroke recovery/rehabilitation and secondary prevention, thereby optimizing our ability to further enhance outcomes after stroke in the not-too-distant future.

*Bruce Ovbiagele* *Bruce Ovbiagele* *Steve Kautz* *Steve Kautz* *Wayne Feng* *Wayne Feng* *DeAnna L. Adkins* *DeAnna L. Adkins*
